# Transanal Extrusion of a Cystoperitoneal Shunt Catheter in a Child: A Case Report and Review of the Literature

**DOI:** 10.7759/cureus.114873

**Published:** 2026-08-20

**Authors:** Dominique Muhindo Balikwisha, Dramane Cissé, Daniel Yamba Yamba, Josué Ilunga Kazayi, Kevin Beya Musangana, Oswald Niyongere, Kassim Balde, Kenza Rahmouni, Oualid Mohammed M Hmamouche, Marouane Hammoud, Lakhdar Fayçal, Benzagmout Mohammed, Chakour Khalid, Chaoui Faiz Mohammed

**Affiliations:** 1 Department of Neurosurgery, Hassan II University Hospital of Fez, Fez, MAR; 2 Department of Surgery, Medpark Clinic, Lubumbashi, COD; 3 Department of Neurosurgery, Sub-Saharan African Future Neurosurgeons Association (SAFNA), Cotonou, BEN; 4 Department of Pediatric Surgery, Hassan II University Hospital of Fez, Fez, MAR

**Keywords:** case report, cystoperitoneal shunt, pediatrics, risk factors, transanal extrusion of catheter

## Abstract

Cystoperitoneal shunt and ventriculoperitoneal shunt are commonly used cerebrospinal fluid diversion procedures in neurosurgery. Although generally effective, these procedures are associated with several complications, including shunt migration. Transanal extrusion of the distal catheter after shunt migration is a rare but potentially serious complication, reported in approximately 0.1%-0.7% of shunt procedures, and is usually associated with bowel perforation and a risk of retrograde central nervous system infection. We report the case of a seven-month-old child who developed transanal extrusion of the distal catheter six months after cystoperitoneal shunt placement for a symptomatic arachnoid cyst. The shunt was successfully removed, and cerebrospinal fluid analysis showed no evidence of infection. Follow-up was favorable, with no recurrence of the arachnoid cyst; therefore, shunt replacement was not required. Here, we discuss the mechanism, predisposing factors, and principles of management.

## Introduction

Arachnoid cysts (ACs) are benign cerebrospinal fluid (CSF)-filled lesions located within the arachnoid membrane. They account for approximately 1% of all intracranial lesions diagnosed in newborns, with a reported incidence ranging from 0.5% to 2.6% [1].

Although most ACs remain asymptomatic and are discovered incidentally, symptomatic lesions may require surgical intervention to relieve mass effect and manage associated neurological signs. Several surgical techniques have been described for the treatment of symptomatic ACs, including microsurgical fenestration, endoscopic fenestration, and cystoperitoneal shunting (CPS) [2,3].

Among these, CPS remains a well-established and effective therapeutic option, providing favorable clinical outcomes across all age groups [2]. However, as with any CSF diversion procedure, CPS is associated with potential complications, including infection and shunt migration [4].

Shunt migration is an uncommon but potentially serious complication, with an overall reported incidence ranging from 0.8% to 3%, irrespective of the indication for shunt placement or the site of migration. Among its various presentations, transanal extrusion of the distal catheter (TEC) is exceptionally rare, occurring in only 0.1% to 0.7% of shunted patients [4-8]. Most cases of TEC following shunt migration reported in the literature have involved ventriculoperitoneal shunts (VPS) [7,8]. Proposed predisposing factors include young age, infection, poor nutritional status, and previous abdominal surgery [6,8]. Although uncommon, this complication may lead to meningitis or ventriculitis, with reported mortality rates approaching 15%, underscoring the importance of early recognition and prompt management [8].

We report a rare case of transanal extrusion of a cystoperitoneal shunt catheter, occurring six months after shunt placement in a seven-month-old child treated for a symptomatic frontoparietal AC. We discuss the possible mechanisms, risk factors, management strategy, and clinical outcome, while reviewing the relevant literature.

## Case presentation

**Figure 1:**
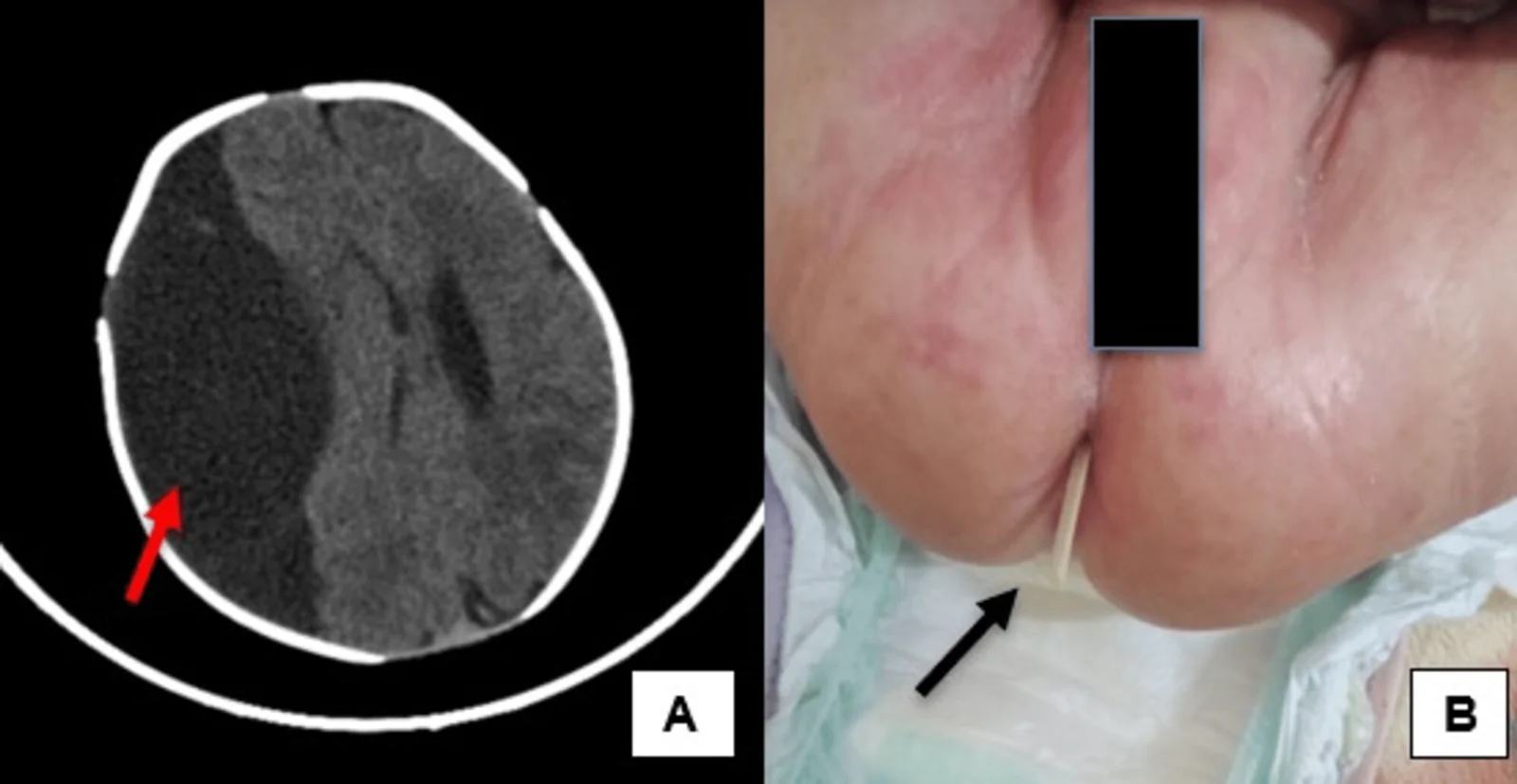
(A) Preoperative brain CT scan obtained at one month of age, demonstrating a right frontoparietal arachnoid cyst (red arrow) causing significant mass effect, with midline shift and compression of the right lateral ventricle. (B) Clinical photograph showing transanal extrusion of the distal end of the cystoperitoneal shunt catheter (black arrow).

A seven-month-old female child was brought to our pediatric emergency department after her mother noticed protrusion of the distal shunt catheter through the anus. At one month of age, the patient had undergone CPS placement for a symptomatic intracranial AC presenting with seizures (Figure 1, Panel A). A medium-pressure Sophysa valve was used, and the postoperative course was uneventful, with complete resolution of the seizures. Six months after shunt placement, the mother incidentally noticed the distal catheter protruding from the anus while bathing the child, prompting immediate medical evaluation.

**Figure 2:**
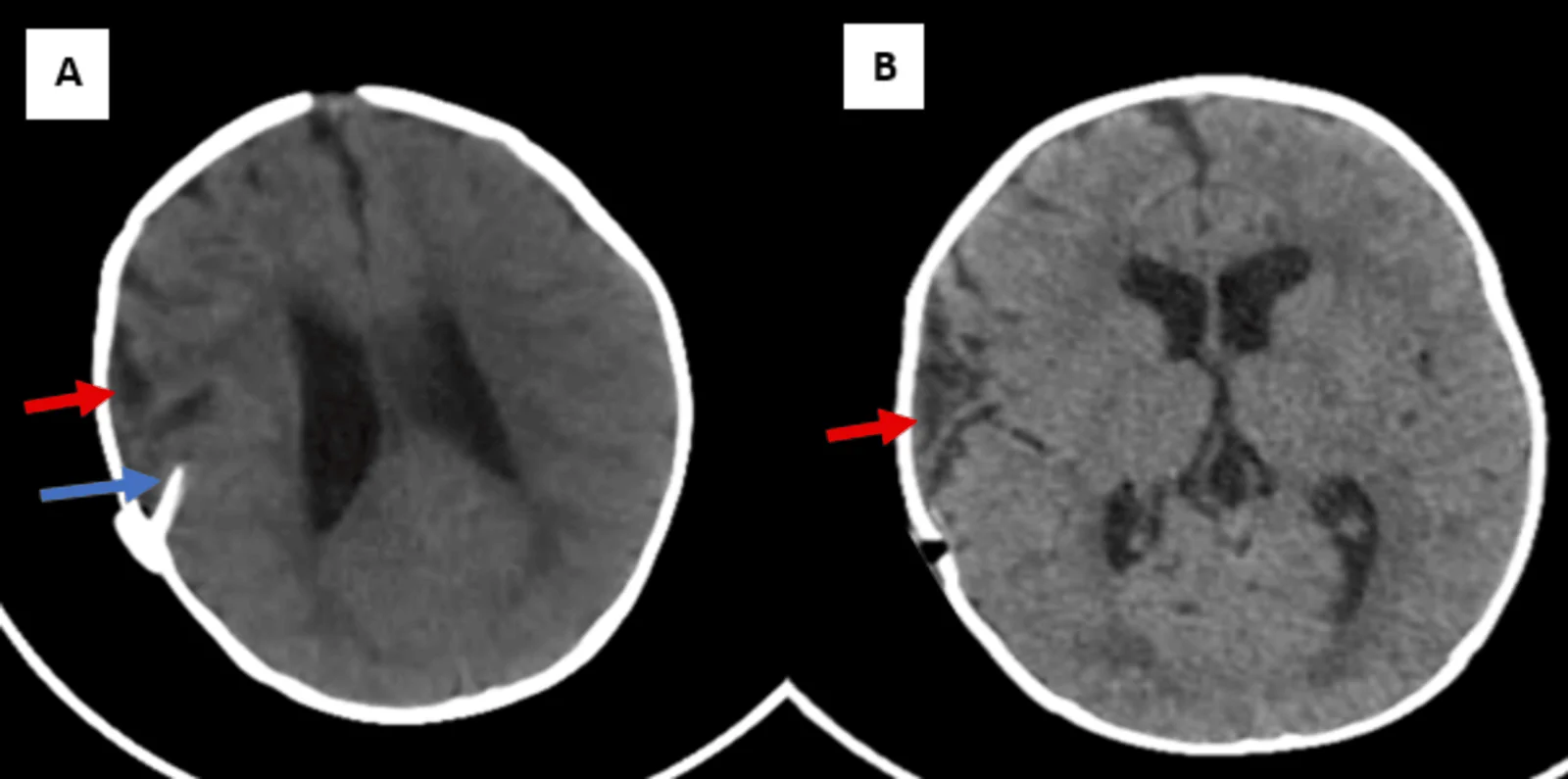
(A) Preoperative brain CT scan obtained after transanal extrusion of the cystoperitoneal shunt, demonstrating the intracranial catheter remaining in its original position (blue arrow) and complete radiological resolution of the arachnoid cyst (red arrow). (B) Follow-up brain CT scan obtained one year after shunt removal, confirming persistent complete resolution of the arachnoid cyst (red arrow) without evidence of recurrence.

**Figure 3:**
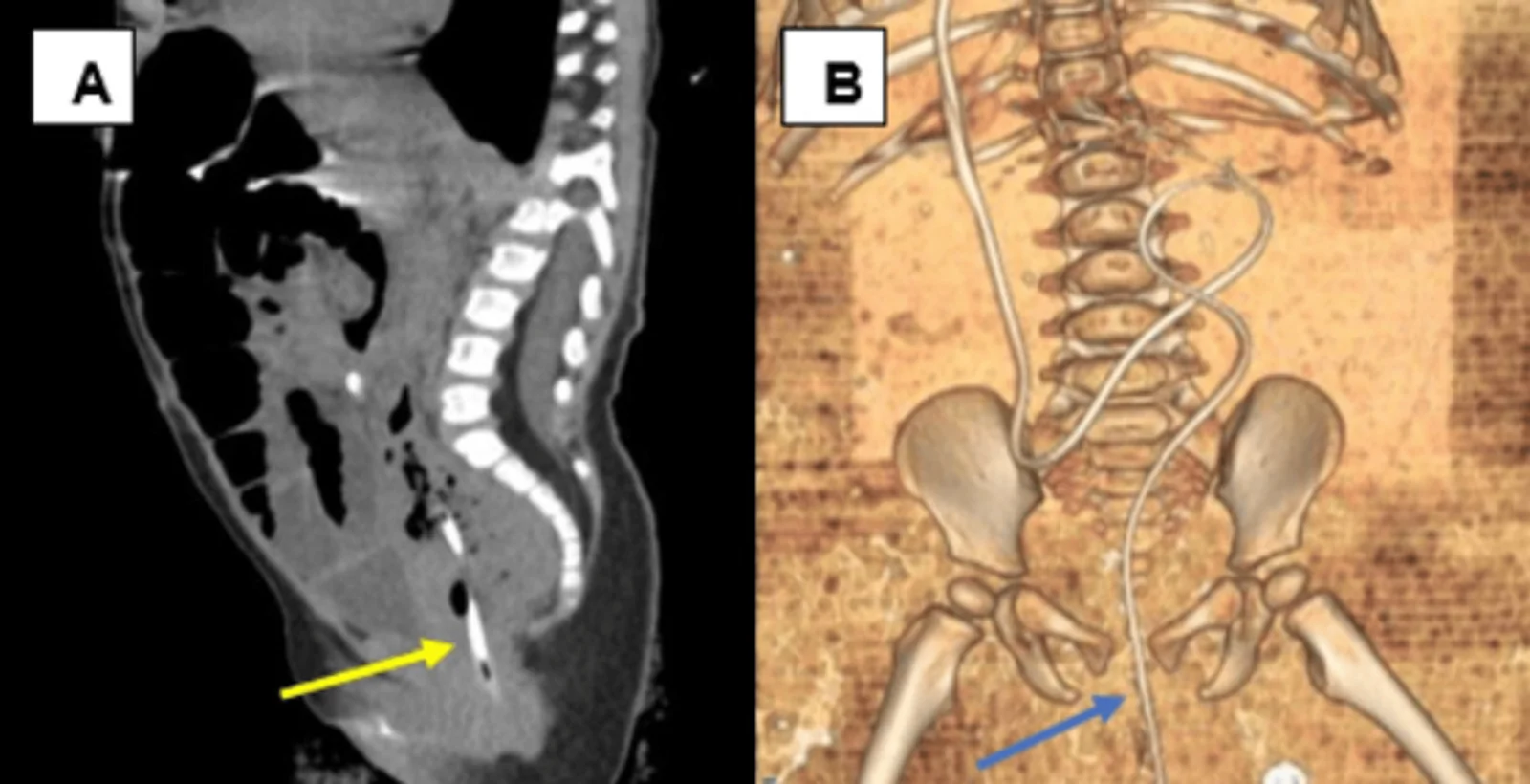
(A) Sagittal abdominal CT scan demonstrating migration of the distal cystoperitoneal catheter (yellow arrow). (B) Three-dimensional CT reconstruction clearly illustrating the course and distal migration of the cystoperitoneal catheter (blue arrow).

On admission, the child was clinically stable and in good general condition, weighing 7.3 kg and measuring 66.3 cm in length. She was afebrile, with no clinical signs of peritoneal irritation. Head circumference was within the normal range for age. The cranial and abdominal surgical wounds were well healed, with no signs of local infection. Neurological examination was normal, and exploration of the perianal region revealed TEC (Figure 1, Panel B). On brain CT scan, the intracranial catheter remained in place, with complete regression of the AC (Figure 2, Panel A). Abdominal CT scan confirmed migration of the distal catheter through the colon, with transanal extrusion, without evidence of an intra-abdominal abscess or CSF pseudocyst (Figure 3).

**Figure 4:**
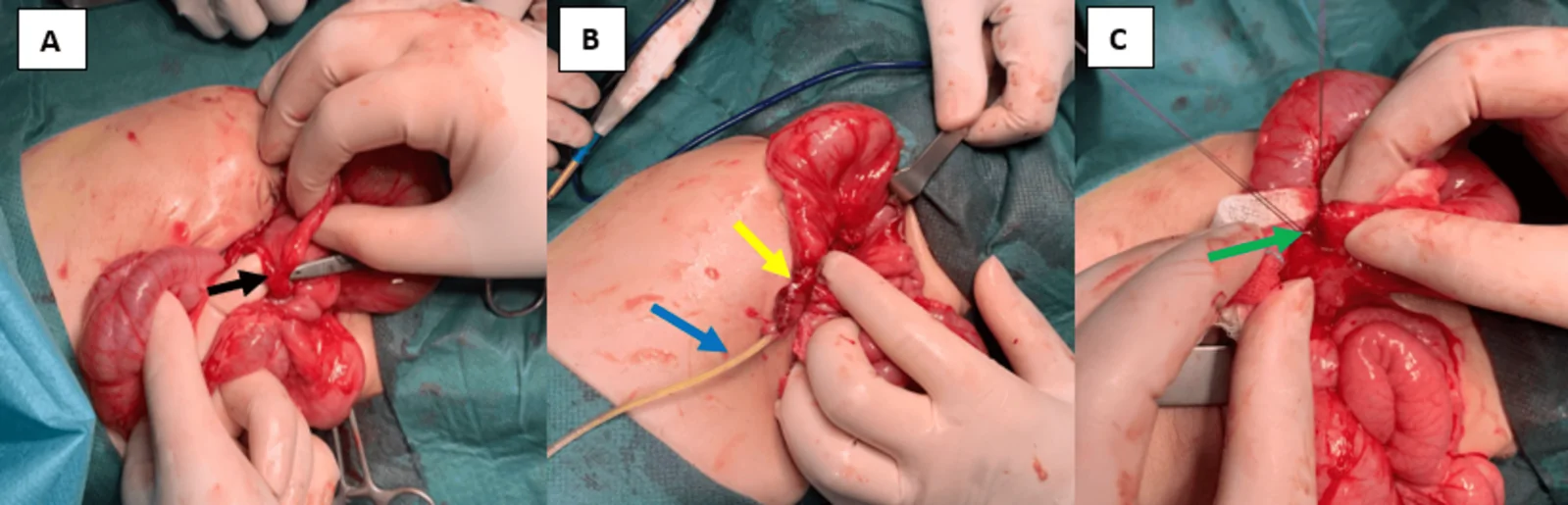
Intraoperative findings during surgical management. (A) Dense fibrotic tissue surrounding the shunt catheter at the site of perforation (black arrow). (B) Proximal segment of the catheter (blue arrow) embedded within the fibrotic tissue (yellow arrow). (C) Appearance after adhesiolysis, caudal traction, and removal of the catheter, followed by primary closure of the colonic defect with 4-0 Vicryl sutures (green arrow).

Given the potential risk of central nervous system (CNS) infection, complete removal of the shunt system was undertaken through a multidisciplinary approach involving neurosurgical and pediatric surgical teams. During the first stage, the neurosurgical team reopened the previous cranial incision, removed the proximal intracranial catheter, and disconnected it from the distal peritoneal catheter. However, gentle traction failed to retrieve the distal catheter because of significant resistance. Then a mini-laparotomy was subsequently performed by the pediatric surgeons. Exploration revealed perforation of the left colon, where the catheter was densely encased by fibrous adhesions (Figure 4). Careful adhesiolysis allowed complete mobilization of the catheter, which was then successfully removed through the anus by gentle caudal traction. The colonic perforation was repaired with interrupted 4-0 Vicryl sutures.

CSF and blood analysis revealed no evidence of systemic or CNS infection (Table 1). As prophylactic antimicrobial therapy, the patient received intravenous ceftriaxone (80 mg/kg/day) for 48 hours. The postoperative course was favorable, with no procedure-related complications. The patient was discharged home in good clinical condition on postoperative day three.

**Table 1:** Blood and cerebrospinal fluid laboratory findings. RBC: red blood cells; WBC: white blood cells; Hb: hemoglobin; CRP: C-reactive protein; PCT: procalcitonin

Examination	Result	Reference range
Blood count		
RBC	4.79 × 10¹²/L	4.5–5.5 × 10¹²/L
WBC	10.8 × 10⁹/L	4–10 × 10⁹/L
Hb	15 g/dL	13–17 g/dL
Platelets	430 × 10⁹/L	150–450 × 10⁹/L
Cerebrospinal fluid		
Macroscopic appearance	Clear	Clear
RBC	41 cells/µL	0 cells/µL
WBC	1 cell/µL	0–5 cells/µL
Protein	0.28 g/L	0.20–0.40 g/L
Glucose	3.86 mmol/L	2.50–4.00 mmol/L
Lactate	1.73 mmol/L	1.2–2.2 mmol/L
Culture	Negative	Negative
Inflammatory markers		
CRP	15 mg/L	<10 mg/L
PCT	0.02 ng/mL	<0.05 ng/mL

At the one-year follow-up, she remained asymptomatic, with normal neurological development. Follow-up brain CT scan confirmed persistent complete resolution of the AC without recurrence (Figure 2, Panel B). In view of the sustained radiological remission and the absence of neurological symptoms, reinsertion of a CPS was considered unnecessary.

## Discussion

CPS and VPS are among the most commonly performed CSF diversion procedures in neurosurgical practice. Although generally safe and effective, they are associated with various complications, including shunt migration, which occurs more frequently in the pediatric population than in adults. Shunt catheter migration has been reported at a wide range of anatomical sites [9-13]. When associated with gastrointestinal perforation, this rare complication carries a reported mortality rate of up to 15% [8,9]. In the present case, a seven-month-old female child developed transanal extrusion of a CPS six months after surgery for a symptomatic AC. Despite left colonic perforation, she showed no clinical and biological evidence of CNS infection. Moreover, brain CT scan demonstrated complete resolution of the AC, with the intracranial catheter remaining in its original position, allowing definitive shunt removal without the need for reinsertion. To better characterize this uncommon complication and discuss its risk factors and management, we reviewed the English-language literature indexed in PubMed and identified eight previously reported cases of distal shunt migration.

Because shunt migration following CPS is exceedingly rare, most of the available evidence derives from cases involving VPS. Among the eight reported cases identified in the literature, TEC was the most common pattern of migration, accounting for five cases, whereas scrotal, pulmonary, and urethral migration were each described in one patient. A clear predominance of pediatric cases was observed, with six of the eight patients being younger than 15 years. Among the six cases with available temporal data, migration occurred between five days and twelve months after shunt placement; the interval was not specified in the remaining two cases. CNS infectious complications were reported in four patients, including meningitis in three and multiple brain abscesses in one, while three patients had no documented CNS complications and data were unavailable for one. Management generally required removal of the migrated shunt, with additional treatment tailored to the associated complications. Despite the potentially serious consequences of shunt migration, all eight patients ultimately achieved favorable outcomes.

Several mechanisms have been proposed to explain bowel perforation associated with shunt migration, including chronic mechanical pressure from the distal catheter, local foreign-body reaction, and, less commonly, hypersensitivity to silicone [6,8,14]. Sustained contact between the catheter and the bowel wall may promote adhesion formation, progressive erosion, and, ultimately, perforation. In our patient, the dense fibrotic tissue surrounding the catheter at the left colonic perforation site provides intraoperative support for such a chronic local tissue reaction.

Young age appears to be an important predisposing factor. Atallah et al. reported a median age of three months, with 75% of patients being younger than 18 months at the time of shunt placement [14], while Adel et al. found that 57% (26/46) of affected patients had undergone shunt placement during the first year of life [9]. This pediatric predominance has been attributed to greater tissue fragility and increased intestinal motility [10,15].

Other reported risk factors include poor nutritional status, infection, previous abdominal surgery, and technical factors related to shunt placement [9,10,14]. Poor nutrition was notably implicated in the case reported by Alhassan et al. [16]. None of these additional factors were identified in our patient, who had an adequate nutritional status, no evidence of systemic infection, and no history of previous abdominal surgery. In adults, Abode et al. identified obesity (odds ratio: 6.38; 95% confidence interval: 1.16-35.21; p = 0.033) and previous shunt surgery (odds ratio: 2.17; 95% confidence interval: 1.47-3.22; p < 0.001) as independent predictors of migration [6]; however, comparable pediatric data remain scarce.

The timing of migration in our patient was consistent with previous reports, with median intervals ranging from 6.1 to 10 months [14,15]. Notably, Adel et al. reported that shunt migration becomes less frequent beyond 12 months after placement [9].

Migration of the shunt may compromise CSF diversion and cause complications depending on the anatomical site of migration. Transanal extrusion is particularly concerning because of the risk of ascending CNS infection, including meningitis and ventriculitis. Adel et al. reported meningitis in 35% (18/51) of cases [9]. CNS infection occurred in four of the eight cases included in our review. However, more than 40% of shunt migrations may remain asymptomatic [9,14,16,17]. In our patient, no clinical or biological evidence of CNS infection was found, possibly reflecting early recognition and prompt management.

Another notable finding was the complete radiological resolution of the AC. This may have resulted from progressive cyst regression through the shunt before catheter migration. The absence of recurrence following shunt removal suggests that cyst resolution was sustained and that further CSF diversion was no longer required. This finding is particularly noteworthy, as Zhang et al. reported cyst size reduction in 95% of patients following shunting [2].

Management of shunt migration primarily involves removal of the migrated catheter. In our review, seven of the eight patients underwent shunt removal, consistent with Atallah et al., who reported a removal rate of 51.7% [14]. In cases of TEC, distal extraction through the anus after proximal disconnection has been advocated to minimize the risk of retrograde contamination. When simple extraction is unsuccessful, laparoscopic removal may be considered, particularly in the presence of a CSF pseudocyst, whereas peritonitis or intra-abdominal abscess may require minilaparotomy [9,16]. In our patient, attempted traction was unsuccessful, and minilaparotomy was therefore performed because laparoscopic facilities were unavailable.

CSF analysis is essential to assess for CNS infection and guide subsequent management. In shunt-dependent patients, temporary external ventricular drainage may be required, with definitive shunt replacement considered after resolution of infection and documentation of two sterile CSF cultures [4,9,14]. In our case, shunt replacement was unnecessary because the AC had completely resolved and showed no recurrence during follow-up.

**Figure 5:**
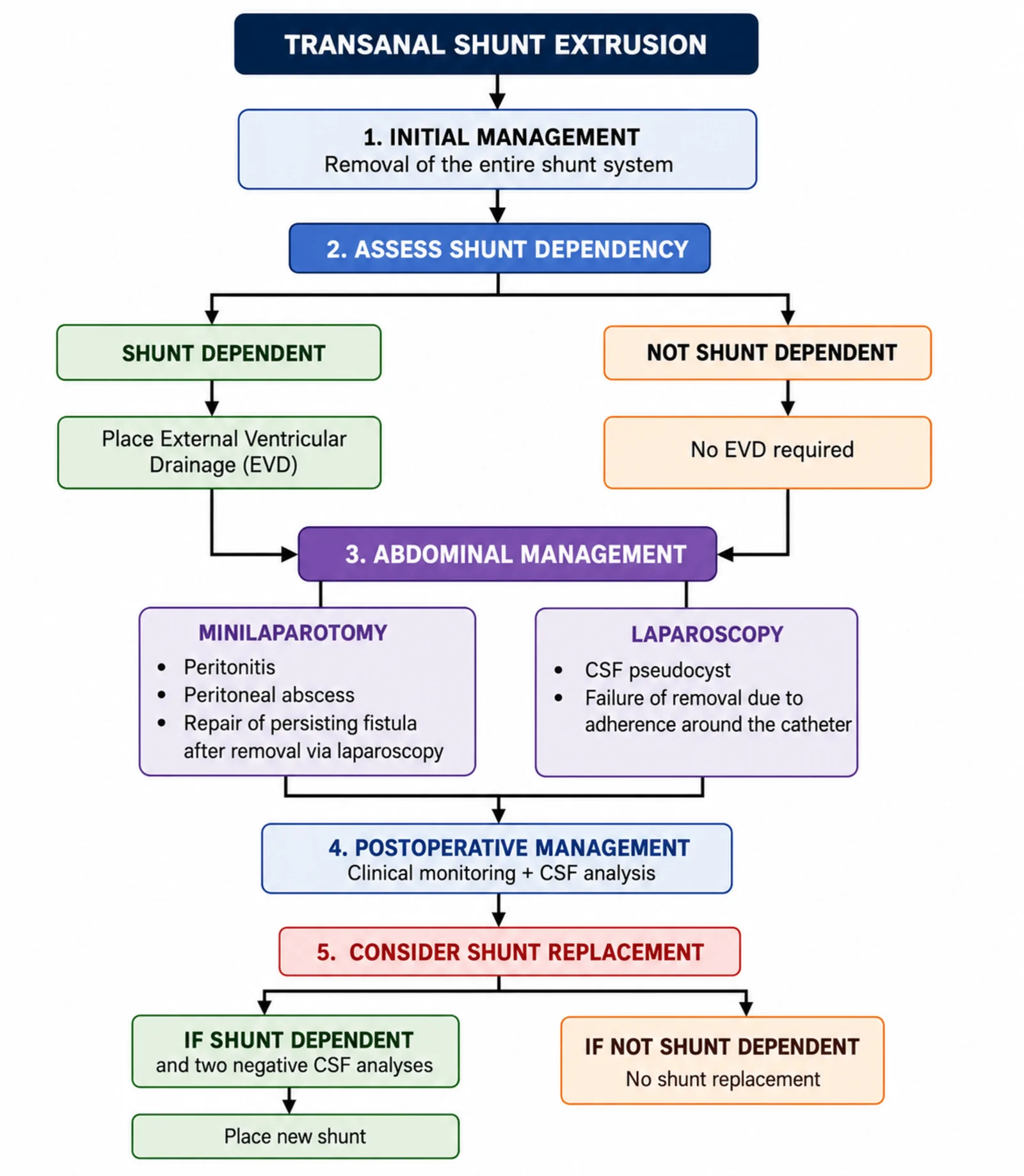
Management algorithm for transanal shunt catheter extrusion.

A comprehensive overview of the previously reported cases of shunt migration is provided in Table 2. Based on these principles, a proposed management algorithm for TEC is presented in Figure 5.

**Table 2:** Literature review summary M: male; F: female; VPS: ventriculoperitoneal shunt; CPS: cystoperitoneal shunt; HCP: hydrocephalus; LMS: lumbar myelomeningocele surgery; CNS: central nervous system; HCP: hydrocephalus

Authors/Year	Case	Age/Sex	Indication of shunt	Type of shunt	Time between migration and shunt placement	Site of migration	CNS complications	Management	Outcome
Basehi et al. (2023) [7]	1	24 months/M	Congenital HCP	VPS	7 months	Anus	Meningitis	Removal of VPS	Good
Kencana et al. (2024) [8]	1	1 month/F	Congenital HCP	VPS	1 month	Anus	No	Removal of VPS	Good
Chanchlani et al. (2023) [11]	1	2 months/-	Congenital HCP	VPS	5 days	Scrotal	No	VPS repositioned	Good
Osman et al. (2017)[12]	1	29 years/F	Congenital HCP	VPS	Not reported	Urethra	No	Removal of VPS	Good
Patel et al. (2022) [13]	1	12 years/M	HCP after LMS	VPS	12 months	Pulmonary artery	Not report	Removal of VPS	Good
Khizar et al. (2022) [15]	1	3.5 years/M	HCP after LMS	VPS	Not reported	Anus	Multiple brain abscesses	Removal of VPS	Good
Alhassan et al. (2020) [16]	1	6 years/M	Congenital HCP	VPS	12 months	Anus	Meningitis	Removal of VPS	Good
Shbani et al. (2024) [17]	1	17 years/M	Post-traumatic HCP	VPS	12 months	Anus	Meningitis	Removal of VPS	Good
Our case (2026)	1	7 months/F	Arachnoid cyst	CPS	6 months	Anus	No	Removal of CPS	Good

Intravenous antibiotic therapy is generally recommended because of the potential risk of retrograde infection, even in the absence of initial CSF abnormalities [16,17]. Early recognition and appropriate management are associated with favorable outcomes, reported in 91.8% (45/49) of patients by Adel et al. and 90% by Atallah et al. [9,14]. In our patient, same-day management following recognition of TEC may have limited the duration of enteric contamination and potentially contributed to the absence of retrograde CNS infection.

The main strength of this report is the description of a rare case of TEC occurring six months after CPS, with a favorable outcome characterized by the absence of retrograde CNS infection and no recurrence of the AC after shunt removal, thereby avoiding the need for shunt replacement. Most cases of shunt migration reported in the literature have occurred following VPS. To our knowledge, only one previous case of shunt migration following CPS has been reported in the Turkish language literature, and that case was complicated by subdural empyema [18]. This report was not included in our review because our literature search was intentionally restricted to English-language publications indexed in PubMed.

An important limitation of our review is the comparison of CPS migration with predominantly ventriculoperitoneal shunt migrations involving various anatomical sites. The mechanisms underlying migration may differ according to initial indication and the site of migration. Nevertheless, the general principles of management remain broadly similar, particularly regarding shunt removal, assessment and treatment of CNS infection, and determination of the need for subsequent shunt replacement.

Further studies are needed to determine whether the risk of shunt migration differs between VPS and CPS. In addition, improvements in shunt system design should be explored to minimize the risk of migration in both types of shunts.

## Conclusions

TEC after CPS or VPS is a rare but potentially serious complication, occurring predominantly in pediatric patients and most often within the first year after shunt placement. Early recognition and prompt shunt removal are essential to reduce the risk of retrograde infection and are generally associated with favorable outcomes. Although most reported cases involve VPS, this complication may also occur after CPS. Further studies are needed to better understand the mechanisms and risk factors associated with CPS migration and to guide the development of shunt systems designed to minimize this complication. Finally, close postoperative surveillance and education of patients’ families regarding warning signs are essential for early detection and timely management.
